# Nanostructured Lipid Carriers for Nose to Brain Delivery Targeting CNS: Diversified Role of Liquid Lipids for Synergistic Action

**DOI:** 10.34172/apb.2022.078

**Published:** 2021-10-04

**Authors:** Avinash Ramrao Tekade, Pradeep Sadanand Mittha, Charushila Sopan Pisal

**Affiliations:** Department of Pharmaceutics, Marathwada Mitra Mandal’s College of Pharmacy, Thergaon, Pune-411033.

**Keywords:** CNS targeting, Blood brain barrier, Intranasal route, NLC, Liquid lipid, Synergistic action

## Abstract

Neurological disorders such as Alzheimer’s disease, Parkinson’s disease, dementia, epilepsy, depression, migraine etc. are affecting more and more elderly people’s day by day. Conventional route of administration to treat these diseases has to face a major hindrance that is blood brain and blood-*cerebrospinal fluid* (CSF) barrier to achieve desired concentration of drug at the site of action for therapeutic effect. Hence, intranasal route of delivery is considered as promising and alternative route to achieve desired goals. In last four decades, brain targeting strategies are widely studied and considered having great potential by researchers; especially intranasal delivery owing to its benefits. Various nano formulations such as nanoemulsions, nanosuspensions, hydrogels, in situ gels, dendrimers and lipidic formulations are studied widely. Lipid nano formulations especially second generation nanostructured lipid carriers offer greater advantages in terms of stability, fabrication techniques, scalability, drug loading and drug targeting. Nanostructured lipid carrier (NLCs) constitute of two major components viz solid lipid and liquid lipid in a specific ratio. In this review, authors have discussed about the possible synergistic actions of oils/liquid lipids with synthetic drugs resulting into great therapeutic benefits.

## Introduction

Neurological disorders are emerging and increasing with great pace. The main hindrances in treating these disorders by conventional route of administration is difficulty in transport of molecules through blood brain barrier and blood-cerebrospinal fluid (CSF) barrier. This results in failure of attaining desired therapeutic concentration in the brain. The blood brain barrier constitutes tight junctions of epithelial cells that doesn’t allow any foreign molecules to reach brain and hence also imposes a significant threat for permeation of drug molecules. Physiological functions of blood-brain barrier (BBB) is to prevent transport of macromolecules, plasma proteins such as albumin, plasminogen and prothrombin as they can damage nervous tissues. Small molecular drugs with lipophilic nature (molecular weight < 400 Da and forms < 8 hydrogen bonds) may cross BBB via lipid mediated free diffusion.^1^ There are many techniques that can be equipped to reengineer the drug molecule to cross BBB such as prodrug method,^2^ and Trojan horse technique for larger sized molecules.^3^ Despite impenetrability of the blood brain barrier, there are pathways by which drugs can transport across BBB such as transcytosis diffusion, carrier mediated transport, transport of ions, receptor mediated transcytosis, and adsorptive mediated transcytosis.^4^

 Despite the presence of complex barrier, there are many proven ways for CNS targeting of drugs for the treatment of various diseases. Mainly, approaches such as noninvasive approach, invasive approach and intranasal route are adopted according to situation by medical practitioner and physician. Invasive approach includes BBB modulation or disruption, intracerebral implants, intracerebral injections/infusions. However, these approaches create discomfort amongst patients and are not preferred as other ways of treatment are available. Noninvasive approaches include use of formulations by conventional oral route. Intranasal route is one of the non-invasive and also a promising approach for CNS targeting and has benefit of, avoiding first pass metabolism and hence therapeutic concentration of drug is attained in minimal dose.

 Nasal mucosal region is considered as a potential route for absorption of molecules because of its highly vascularized epithelium which provides passage for rapid absorption of compounds into systemic circulation thus avoiding hepatic first pass metabolism. Also, lag time associated with oral drug delivery system and metabolic activity in nasal environment is minimal as compared to GIT system, providing benefit over conventional route of administration. The absorption of molecules occurs directly through trigeminal and olfactory nerve pathway, which provides direct entry into brain thus bypassing CNS barrier hurdle, making this route a potential route of administration of the CNS acting drugs.

Hydrophilic drugs and macromolecules such as peptides, proteins, and vaccines are too large to penetrate BBB and also possess the risk of degradation in systemic circulation and in gastric/liver enzymes.^5^ In such cases, drugs incorporated in the form of nanoformulation, administered through intranasal route, can achieve the desired therapeutic levels at the site of action rather than drugs administered through conventional routes or other routes. Besides having numerous advantages over other routes of administration, this route also has some limitations and restrictions for formulating a dosage form for small dose drugs and limited residence time due to fast mucociliary clearance rate.

 This review article gives overview of mechanism of drug transport to brain through intranasal route and potential of nanostructured lipid carriers (NLCs) as a drug delivery system for CNS targeting. The main focus of this review article is on the use of various liquid lipids and their potential for obtaining synergistic effect with synthetic drugs.

## Anatomy of nose

 In the present article an overview of general anatomy of nose is briefly discussed and the emphasis is given on important pathways responsible for transport of drug directly to the CNS.

## Nasal cavity and its important functions

Human nose is structurally divided into two cavities by nasal septum. Average size of human nasal cavity is 12 cm to 14 cm long, and has a surface of around 140 cm^2^-150 cm^2^ and 12-14 mL capacity.^6,7^ Both the nasal cavities are divided into three regions each, namely vestibule region at the front side or at opening of nose cavity and just inside the nostrils, respiratory region present at mid of cavity and olfactory region present at backend. The nasal vestibule has no absorption function. The respiratory region contains three sub imaginary regions i.e. the superior, the middle and the inferior regions, having their important role in producing turbulence for the inhaled air thereby maintaining the temperature of inhaled air to body temperature, filtering the air for clearance of micro particles, microorganisms and dust particles. The olfactory region is located at the roof of the nasal cavity in humans.^5,8,9^ The mucus layer has unique mucociliary clearance (maximum time is 20 minutes) mechanism that gradually transports such particulates to the back of the throat, down the esophagus, and further into the gastrointestinal tract.^5,9-11^ Nasal mucosa also has the enzymes that have metabolic capability of converting endogenous materials into compounds that are eliminated more readily.

## Respiratory epithelium

Basically respiratory epithelium is composed mainly of four types of cells, (a) goblet cells (functions by secretion of mucus), (b) ciliated cells, (c) non-ciliated cells and (d) basal cells. Active transport process such as exchange ions and water in between the cells is facilitated by these cells. Cilia also plays an important role of mobilization of molecules and maintaining moisture content there by avoiding drying of mucus layer.^5,8^ Mucus is secreted by goblet cells, which is complex mixture containing 95% water, 2% mucin, 1% salts, 1% other materials (Proteins such as immunoglobulins, albumin, lysozymes, enzymes, lipids, etc.).^5^

The nature of respiratory epithelium is highly vascularized and contains a large number of blood capillaries present in mucosal region. These blood capillaries are site of targets for drug absorption through nasal route. Formulations are designed in such a way that they get firmly adhered to mucosal region and hence increasing the therapeutic level of drug at the target site.^8,9^

## Olfactory epithelium

 Structurally epithelial layer of the olfactory region mainly consists of three types of cells i.e. (a) Olfactory neuronal cells, (b) sustentacular cells (supporting cells) and (c) basal cells.

The olfactory neural cells, or axons, are unmyelinated cells and interspaced between supporting cells. They originate at the olfactory bulb and extend upto the apical surface of the olfactory neuroepithelium.^5,8^

Basal cells are mainly progenitor cells (of supporting cells) that acts by providing mechanical support by anchorage mechanism to other cells.^12^

## Mechanism of drug transport to brain when administered through nasal route

Drug delivery across the nasal cavity takes place through two main pathways (Respiratory region and Olfactory area). The former region is well vascularized while the later one has olfactory neurons exposed in upper area of nares. Transport of drug compounds through respiratory area occurs by nasal epithelium which is of vascularized nature and mainly via trigeminal nerves. Transport of molecules from olfactory region occurs through olfactory neurons by transcellular mechanism across olfactory bulb, that has direct passage to the brain.^5,8,9^

## Limitations

 Many limitations arise while formulating a formulation targeting directly to CNS via nasal route. Some of the limitations that are considered very important for formulator while designing a formulation is volume of dose in case of liquid formulation, size of dose for powdered formulation hence, potent drugs preferred, some drugs get degrade by enzymes present in nasal cavity and the drug-excipient stability is important.

One of the key aspects for developing nose to brain drug delivery system is their safety and toxicological assessment. The extended contact of mucoadhesive formulation with nasal mucosa may lead to irritation, tissue damage, epithelial toxicity or ciliotoxicity and may result in development of environment friendly for microbial growth.^6,13^

## Nano formulations/nanoparticulate formulations

Drug targeting to human brain has always been challenging for formulators due to the presence of strong barriers such as blood brain barrier and blood cerebrospinal fluid barrier as discussed earlier. After intensive research, intranasal route has been found as a potential route for drug transport directly to brain. Drug delivery through nasal route is considered as non-invasive route.^1^ The ability of nasal mucosal layer to transport the small sized molecules has also been explored widely. Various drug delivery technologies have been emerged, amongst them nanoparticulate system is fascinating part for formulation and a major area of interest due to its potential benefits like ease of preparation, long term stability, achieving desired therapeutic concentration at the site of action, drugs can encapsulated and protection from environmental degradation.^14,15^

 Recent trend in the field of formulation and development has seen an exponential increase in preparation of nano formulations and nanoparticles for drug delivery at desired site of action. Nano formulation having size range of 10 nm-1000 nm has potential to reach at the site of action due to nano sized benefit. Nanoparticulate formulations are engineered by utilizing biodegradable polymers incorporating the drug inside the core structure.

Having the advantage of small size, the potent drugs are easily incorporated in the form of nanoparticles and can be delivered directly to the CNS through intranasal delivery route through respiratory epithelium or olfactory neurons. Nano formulations are engineered in various forms such as polymeric nanoparticles, liposomes, solid lipid nanoparticles, niosomes, ethosomes, dendrimers, microemulsion, nanoemulsion, cubosomes, hydrogels, aquasomes, nanostructured lipid carrier, nanoparticles incorporated in the form in situ gel.^16,17^ These formulations pass directly through the intranasal pathways to CNS either by cellular pathways or through mucosal adhesion in case of mucoadhesive formulations. Nano formulations have also been used in optical imaging technology.^18^ Wais et al discussed commonly used various techniques for production of nanoparticles.^19^ Further discussion in the present review article is regarding various types of lipidic nanoparticles along with their detailed explanations.^20^

## Liposomes

Liposomes are spherical shaped lipid vesicles ranging from few nanometers to several micrometers, however considering its medical use in terms of nano formulation the optimum size range must be somewhere between 10 nm to 500 nm. Liposomes were initially discovered in the 1960s by Alec Bangham at the Babraham Institute, University of Cambridge and consist of single/double/multiple lipid bilayers encapsulating as aqueous compartment.^21-23^

Liposomes are capable of encapsulating both hydrophilic and lipophilic drug molecule in the lipid membrane and aqueous core respectively.^24-26^

## Physicochemical characteristics of liposomes and its composition

The importance of liposomes as a carrier system strictly depends upon the nature of components, size, surface charge and lipid content. Formulation of liposomes mainly contains phospholipids, amphiphilic molecules that have a hydrophilic head and two non-polar hydrophobic chains. When phospholipids dispersed in aqueous medium, they have strong tendency to form membranes due to their amphipathic nature. While their polar heads interact with the aqueous environment and, their long non-polar aliphatic chains promote interaction with one another.^27^

Liposomes are generally classified on the basis of Size such as (a) small, (b) intermediate and (c) large and on the basis of lamellarity they are (a) Unilamellar, (b) oligolamellar, (c) multilamellar vesicles.^27,28^

Generally, liposomes are fabricated by hand shaking method, sonication method, freeze dried rehydration method, reverse phase evaporation method, thin-film hydration or Bangham method and solvent injection method.^28,29^

Liposomal formulation targeting CNS is widely studied in last 5 decades. The obtained results for liposomes targeting CNS through intranasal route shows optimum therapeutic concentration required for treating disease. Some of the optimized liposomal formulations have been summarized in Table 1.

**Table 1:** Summary of optimized liposomes formulated using different lipids

**Drug**	**Disease targeted**	**Lipids used**	**Method used**	**Reference**
Acyclovir	Herpes Simplex Virus infection	DPPC:CHOL(1.6:6)	Thin film hydration technique	^30^
Ghrelin	Cachexia	Cholesterol:Lipoid S100(50:50)	Lipid film rehydration technique	^31^
Risperidone	Schizophrenia	Soya phosphatidylcholine (SPC): Cholesterol	Thin film hydration method	^32^
Rivastigmine	Alzheimer’s.	Egg phosphatidylcholine (EPC): Cholesterol (1:1)	Ammonium sulfate gradient loading method	^33^

## Solid lipid nanoparticles (SLNs)

SLNs are another type of lipid nanocarrier system consisting of hydrophobic core in the range of nanometers.^34,35^ The solid hydrophobic core contains monolayer of phospholipid coating, having drug dissolved or dispersed in the solid matrix. Hence, they have capability to carry hydrophilic or lipophilic drugs.^36-38^ The lipids used as hydrophobic core are biocompatible and its nature is similar as that of human biological membrane, thus additional advantage is gained over other formulations for selecting drug delivery system. These lipids further get degrade after its systemic administration thus, avoiding toxicity.^39,40^

## Essential components of SLN

###  Solid lipids

The selection of solid lipid is a critical factor in SLN fabrication. The melting point of selected solid lipid must be anywhere around 50-80°C or above room temperature. The different class of lipids used for the preparation of SLN are partial glycerides, triacylglyceride (e.g., tristearin, tripalmitin) and their mixtures (e.g., mono-, di- and tri-esters of glycerol and behenic acid), fatty acids (e.g., stearic acid, Oleic acid), steroids (e.g., cholesterol), fatty alcohol (e.g., cetyl alcohol), non-glyceride esters of saturated fatty acids with saturated fatty alcohols (e.g., cetyl palmitate, cetostearyl Palmitate ) and waxes.^41-43^

## Surfactants

Surfactants are the substances that have a major role in fabrication of all the pharmaceutical formulation. Surfactants contribute by decreasing the surface tension between hydrophilic and lipophilic components and thereby providing a stable formulation. Surfactants possess various functional groups, having solubility in either aqueous phase or oil phase. Thus, surfactants with hydrophobic group have affinity towards lipophilic phase and surfactants with hydrophilic group have affinity towards aqueous phase. In general, surfactants are amphiphilic in nature. Depending on the number of functional groups present, their affinity changes towards specific phase. Surfactants are also classified depending on their ionic and nonionic nature. Ionic surfactants have further sub-classification such as anionic surfactant (eg, sodium taurocholate, sodium cholate, sodium glycocholate, sodium lauryl sulphate), cationic surfactant (eg, Stearyl amine, alkyltrimethylammonium bromide) and non ionic or amphoteric surfactant (eg, phosphatidylcholine, polysorbate 60, sorbitan palmitate, poloxamer 407, sorbitan stearate, polysorbate 80, poloxamer 188, alkyl polyglucosides).^41-43^ These surfactants dissociates into ions depending on the pH of the medium.

## Stabilizers, preservatives

 Stabilizers play important role to keep the formulation stable for long duration of time without any particulate aggregation due to surface charge built on it. Generally major phase in SLN constitutes of aqueous phase. This can lead to microbial growth during long storage. Hence for the prevention of microbial growth, preservatives are added (eg, benzalkonium chloride).

## Application

SLNs started to gain attention as a lipidic formulation in 1990s. From then, the tremendous research wok has been done and various routes of administration have been explored for administration of SLN such as topical delivery, oral delivery and intranasal route for CNS targeting. Some of the recent work in developing and optimizing SLN are summarized in Table 2.

**Table 2:** Summary of optimized SLN preparation having different lipids

**Drug**	**Disease targeted**	**Lipids used**	**Method used**	**Reference**
Rosmarinic acid	Huntington’s disease	Glyceryl monostearate	Hot homogenization technique	^44^
streptomycin	tuberculosis	Compritol ATO 888	Patented nano colloidal aqueous dispersion technique	^45^
rivastigmine	Alzheimer’s.	Compritol ATO 888	Homogenization and ultrasonication method	^46^
Carvedilol	Hypertension	Precirol		^47^
Agomelatine	Depression	Gelucire 43/01.	Emulsification solvent evaporation technique	^48^

## Nanostructured lipid carriers

Nanostructured lipid nanoparticles are modified version and next generation SLNs. These drug delivery systems were introduced in order to overcome the possible difficulties of SLNs. NLCs have the major advantage over SLNs such as increased loading capacity, stability during storage, also prevents drug expulsion during long term storage and less water content.^49-52^

## Composition of nanostructured lipid carriers

NLCs are a binary mixture of solid lipids (fats) and liquid lipids (oils) at ambient temperature. Concentration of solid lipid and liquid lipid in the formulation generally ranges from 50:50 upto 90:10.^53,54^ Surfactants included in preparation are in the range of 1-5%(w/v). Surfactants have major role in stability of NLC as well as preparation of stable formulation by decreasing surface tension between lipid phase and aqueous phase.^55^ Drug is loaded in liquid lipids and then liquid lipid is loaded in solid lipids and hence double protection is provided in the form of core structure from external degradation factor. Selection of solid lipids and liquid lipids play an important role in stability of NLCs for long term use. All the components used for production of nanostructured lipid carrier must comply with the regulatory agencies as GRAS (generally recognized as safe).

## Advantages of NLCs

 Physical stability is improved as compared to SLN. Dispersion in aqueous phase is increased and hence observed high entrapment efficiency of hydrophilic drugs and lipophilic drugs. Particle sizes are controlled and the NLC showed better penetration ability. Use of organic solvents in production of NLC is avoided as in the case of preparation of other nanoparticulate systems. NLCs are prepared with lipids which are biodegradable, well tolerated and easily thrown out of the body.

 Depending upon the various available production methods and the different concentration of lipids, different types of NLCs are obtained.

## Various types of nanostructured lipid carriers

###  The imperfect type NLC (type 1)

Imperfect type of NLC have disordered shaped solid matrix. Imperfect shape is caused due to incorporation of a fraction of solid lipid by liquid lipid (or oil). This results in small voids formation. Thus, this phenomenon leads to availability of extra space for accommodating drug molecule and gives higher drug pay load. Use of minute quantity of glycerides can overcome this situation. Hence, formation of imperfect shapes provides extra space for drug loading, avoiding formation of highly structured and ordered matrix which would have expelled drug out of the core.^56^

###  The amorphous type NLC (type 2)

For formation of amorphous form of NLC, incorporating solid lipid which remains in alpha polymorph after solidification and storage along with liquid lipid/oils gives amorphous core. The beta polymorph form of solid lipid gives crystalline core/matrix. This type of NLC gives more advantage as no crystalline structure is forced and hence drug remains embedded in the core.^57^

###  The multiple type NLC (type 3)

This type of NLC is basically fat-in-water or oil-in-solid, which can be developed only by phase separation method. Drugs showing higher solubility in oil/liquid lipid than multiple type of NLC are preferable for production by utilizing phase separation technique. Hence it improves drug loading capacity and stability. Tiny droplets of oils are dispersed in solid lipid a then dispersed in aqueous phase. Phase separation technique is further discussed below in the section of methods of production of NLC.^58^

## Preparation techniques of NLCS

 Following are the various production techniques briefly explained:

## High pressure homogenization (HPH)

HPH method is highly reliable and powerful technique for large scale production of NLCs. By utilizing HPH process a stable formulation is obtain with desired nano sized particles. As the name suggests, high pressure (100-2000 bar) is applied resulting into shear stress and thus breakdown of microsized particles into nanosize. Depending upon the desired size of particles, various cycles are performed (10 000 rpm, 800 bar with 10-12 cycles). For obtaining nanoparticles both the phases viz. aqueous and lipid phase has to homogenize at equal temperature. Hot and cold high pressure homogenization technique can be employed to prepare NLC.^57,58^

## Emulsification ultrasonication technique

Method of preparation of NLC by emulsification ultrasonication technique is identical to high pressure homogenization. Solid lipid, liquid lipid and drug are melted at approximately 10°C above melting point of solid lipid. Aqueous phase contains surfactant, co-surfactant and other excipients and heated. By maintaining the same temperature of both the phases, aqueous phase is added drop wise into lipid phase and this pre-emulsion is homogenized. The same pre-emulsion is subjected to ultrasonication for specific time and then added to specified volume of water. This mixture is cooled down to room temperature to obtain NLCs.^56,59^

## Solvent emulsification-evaporation technique

This method incorporates the use of water immiscible organic solvent to dissolve lipophilic material and hydrophobic drug using high speed homogenizer. Further, organic solvent is evaporated by either mild heating or mechanical stirring at room temperature.^55^

## Solvent diffusion

This technique utilizes water miscible solvents such as ethanol, and methanol to dissolve drug and lipid either in mixture of solvent or single solvent. Organic phase is then added to aqueous phase containing pre-dissolved surfactants, stabilizers and other excipients at same temperature under mechanical stirring. Further the mixture is cooled to room temperature for evaporation of organic phase.^59^

## Solvent injection

Basically, in this method lipids are dissolved in water-miscible solvent and then added to aqueous phase by the use of fine needle injection. The main advantage of using this mixture is avoidance of highly sophisticated mixture like high pressure homogenizer and probe sonicator.^56^

## Phase inversion techniques

This technique is based on two steps. The phenomenon behind this technique is first temperature of the mixture is increased at certain temperature, and then decreased by 20-30°C and again elevated to previous temperature. Then finally irreversible shock is induced by cold temperature (0°C). Phase inversion technique is a cumbersome method.^59^

## Applications

Recent trend showed increasing interest of researchers towards second generation nanoparticles i.e. nanostructured lipid carriers. NLC is considered as stable formulation from the past established work by the researchers. Table 3 summarizes the work reported by researchers on NLCs, proving the benefits over other lipid carriers.

**Table 3:** Summary of NLC formulations prepared using different lipids

**Drug**	**Disease targeted**	**Lipids used**	**Method used**	**Reference**
Sumatriptan	Migraine	Stearic acid: Triolein	Solvent diffusion evaporation technique	^60^
Teriflunomide	Multiple sclerosis	Compritol 888 ATO: Maisine 35-1	Melt emulsification ultrasonication method	^61^
Ziprasidone	Schizophrenia	Gelucire 43/01:Capmul MCM	Hot homogenization and Ultra sonication	^62^

 Since liquid lipid is an essential component of Nanostructured lipid carrier system, it has to be explored and studied in depth for obtaining its possible synergistic action. The liquid lipids are basically composed of fatty acids, triglycerides, monoglycerides etc. Oils of natural source obtained from extraction of seeds, bark, leaves etc. with therapeutic value can be incorporated in fabrication of NLC. The present review article emphasizes on the oils obtained from natural source along with established therapeutic activity.

## Olive oil

The composition of olive oil is primarily triacylglycerols (~99%) and secondarily free fatty acids, mono- and diacylglycerols, and an array of lipids such as hydrocarbons, sterols, aliphatic alcohols, tocopherols, and pigments. A plethora of phenolic and volatile compounds are also present. Some of these compounds contribute to the unique character of the oil.^63^

## Hydrocarbons

Squalene and β-carotene (pigment) are the major two hydrocarbons present in olive oil. The last metabolite preceding in sterol ring formation is squalene (2,6,10,15,19,23- hexamethyl-2,6,10,14,18,22-tetracosahexaene) which also partially responsible for health benefit and chemoprotective action against certain cancer.^63^

Oleocanthal is a phenylethanoid and a type of natural phenolic compound found in extra virgin olive oil.^64^ The recent study on extra virgin olive oil rich in oleocanthal showed enhanced effect of donepezil by reducing amyloid-β load in the treatment of Alzheimer’s disease in a mouse model.^65^

## Castor oil

The castor oil has been used traditionally from ancient times for its potential benefit. The major constituent of castor oil is 90% ricinoleic, 4% linoleic, 3% oleic, 1% stearic, and less than 1% linolenic fatty acids. The reason for the use of oil is due to presence of ricinoleic acid in highest amount. But the amount of ricinoleic acid present in the seed oil depends on the cultivation technique, harvesting technique, extraction technique and the region of cultivation.^66^ The hydroxyl functionality of ricinoleic acid makes it a polyol giving it a oxidative stability and a rela­tively high shelf life compared to other oils.^67^ The experimental study showed that ricinoleic acid induces laxation and uterus contraction by activating prostaglandin EP3 receptor.^68^ Experimental model have proven ricinoleic acid has analgesic and anti-inflammatory activity.^69^ Thus, these two activities can be explored for treatment of various brain disorders along with the effect of drug for synergistic effect in the form of NLC.

## Rapeseed oil

Rapeseed oil contains 18 carbon unsaturated acid, bioactive compounds and essential source of unsaturated fatty acids from n-6 and n-3 groups. These compounds have primarily anti-oxidant activity.^70^

## Lavender oil

Phytochemical studies of extract of lavender oil revealed that the major constituents are endo-borneol, 1,8- cineole and compounds those are found in minor quantity are limonene, terpinen-4-ol, -pinene, camphene, p-cymene and cryptone.^71^ The studies have been performed for various therapeutic and pharmacological activities such as anticonvulsant, anxiolytic, anti-inflammatory and antioxidant.^72^

Table 4 summarizes various other oils from natural origin having pharmacological activity.

**Table 4:** Examples of oils which may have potential synergistic action with synthetic drugs

**Oil**	**Activity**	**Reference**
Chamomile	Treatment of anxiety and depression	^73-75^
Eucalyptus oil	Anti-activity, anti-oxidant activity, anti-microbial activity, antifungal, anti-inflammatory	^76,77^
Oregano oil	Anti-oxidant, anti-inflammatory, anti-bacterial	^78^
Lemon grass oil	Anti-inflammatory and sedative activity	^79^
Cumin seed oil	Anti-inflammatory, anti-oxidant and anti-cancer activity	^80,81^
Clove oil	Antimicrobial, antioxidant, antifungal, antiviral and anaesthetic activity	^82,83^
Thyme oil	Antioxidant, sedative property, antitumor and antimicrobial action	^84,85^

## Conclusion

 Numerous nanoparticulate drug delivery systems have been explored widely in laboratory at academic levels. The lipids used in NLC formulation are biocompatible, biodegradable, easily available and most importantly these are approved as GRAS status. Large scale production and scalability of NLC’s is not an issue as simple technique is used for production such as high-pressure homogenization. The liquid lipids (Natural oils) incorporated in fabrication of NLCs play important role treating disorder efficiently due to their synergistic action with active pharmaceutical ingredients.

## Ethical issues

 Not applicable

## Conflict of interest

 None.
